# Supplementary material for: The Nature and Quality of Australian Supermarkets’ Policies That Can Impact Public Health Nutrition, and Evidence of Their Practical Application: A Cross-Sectional Study
Source: Nutrients. 2019 Apr 15;11(4):853. doi: 10.3390/nu11040853 (PMC6520865; doi:10.3390/nu11040853)
Supplement: Supplementary file 1 [file nutrients-11-00853-s001.pdf]

Table S1: Supermarket own brands present on foods and non-alcoholic beverages in three Australian supermarkets

Supermarket own brand present	Frequency	Percent
Coles own brands		
Coles	1482	85.6%
The Bakery at Coles	93	5.4%
Coles Simply Gluten Free	47	2.7%
Coles Organic	28	1.6%
Coles Smart Buy	22	1.3%
Coles Finest	14	0.8%
Coles Grill	12	0.7%
Coles Simply	11	0.6%
Coles Make at Home	8	0.5%
Coles Bake at Home	7	0.4%
Coles Gluten Free	4	0.2%
Coles Simply Less	3	0.2%
Total Coles audited products	***1731***	
Woolworths own brands		
Woolworths Select	631	34.8%
Woolworths	506	27.9%
Woolworths Homebrand	153	8.4%
Macro Organic	114	6.3%
Macro	81	4.5%
Woolworths in-store bakery label	80	4.4%
Woolworths Essentials	54	3.0%
Woolworths Gold	46	2.5%
Woolworths Free From	32	1.8%
Woolworths Created with Jamie	15	0.8%
Jamie Oliver	14	0.8%
Macro Free Range	14	0.8%
Baby Macro Organic	12	0.7%
Woolworths Market Value	12	0.7%
Woolworths Delicious Nutritious	10	0.6%
Woolworths The Odd Bunch	8	0.4%
Woolworths Simply Roast	7	0.4%
Woolworths Select Once Upon a Time	6	0.3%
Farmer's Own	5	0.3%
Mini Macro	4	0.2%
Woolworths Simply Heat	4	0.2%
Gold	1	0.1%
Macro Grass Fed	1	0.1%
Woolworths Simply Cook	1	0.1%
Woolworths Simply Stir-fry	1	0.1%
Total Woolworths audited products	***1812***	
IGA own brands		
Black & Gold	273	68.8%
SUPA IGA	49	12.3%
IGA Baker's Oven	38	9.6%
IGA Signature	24	6.0%
Community Co	12	3.0%
IGA Signature AWARE	1	0.3%
Total IGA audited products	***397***	
**Total audited products**	**3940**	
